# The Potential of 3D Printing in Thermal Insulating Composite Materials—Experimental Determination of the Impact of the Geometry on Thermal Resistance

**DOI:** 10.3390/ma17051202

**Published:** 2024-03-05

**Authors:** Beata Anwajler, Jerzy Szołomicki, Paweł Noszczyk, Michał Baryś

**Affiliations:** 1 Faculty of Mechanical and Power Engineering, Wroclaw University of Science and Technology, 50-370 Wroclaw, Poland; beata.anwajler@pwr.edu.pl; 2 Faculty of Civil Engineering, Wroclaw University of Science and Technology, 50-370 Wroclaw, Poland; pawel.noszczyk@pwr.edu.pl; 3 Energia Investa, 55-114 Szewce, Poland; energiainvesta@gmail.com

**Keywords:** 3D printing, AM technology, thermal insulation, cellular composites, cellular structure, TPMS, gyroid structure, Kelvin cell, cold storage

## Abstract

This paper focuses on the analysis of the thermal properties of prototype insulation structures produced using SLS and SLA additive technologies. There is a noticeable lack of analysis in the scientific literature regarding the geometry of 3D-printed structures in terms of their thermal properties. The aim of this paper was to analyze printed samples of prototype thermal insulation composite structures and their potential for use in building applications. The research material consisted of closed and open cell foams of varying structural complexity. Increasing the complexity of the composite core structure resulted in a statistically significant decrease in the value of the thermal conductivity coefficient λ and the heat transfer coefficient U, and an increase in the thermal resistance Rc. The experimental results showed that the geometric structure of the air voids in the material is a key factor in regulating heat transfer. The control of porosity in materials produced by additive technology can be an effective tool for designing structures with high insulation efficiency. The best performance of the prototype materials produced by the SLS method was a three-layer cellular composite with a gyroid core structure. It was also shown that the four-layer gyroid structure panels with an outer layer of metallized polyethylene film produced using 3D SLA printing had the best thermal insulation. As a result, the analysis confirmed the possibility of producing energy-efficient insulation materials using 3D printing. These materials can be used successfully in construction and other industries. Further research will significantly improve the quality, accuracy, and speed of printing insulation materials, reduce the negative impact on the natural environment, and develop intelligent adaptive solutions.

## 1. Introduction

**Figure 1:**
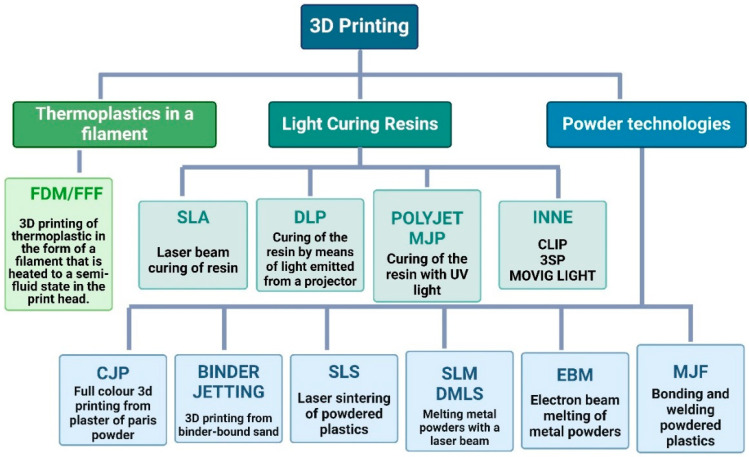
Division of incremental technologies (elaborated by authors).

With the modern trend towards efficient energy management, effective thermal insulation is an important element in its implementation. Scientific research is currently focused on finding innovative insulation structures and producing them using the latest manufacturing methods. One of the most advanced methods is additive manufacturing (AM), also known as 3D printing. The ability to create complex geometries, adapt designs, and use advanced materials creates opportunities for more efficient and stable heat transfer solutions. One of the key benefits of additive technologies is the potential reduction in material waste compared to traditional manufacturing methods [1]. By optimizing the design and structure of heat transfer components, 3D printing enables the creation of lighter yet more efficient solutions and systems [2]. Localized component manufacturing reduces the need for intensive transportation and associated carbon emissions. Emissions can lead to reduced energy consumption and improved overall energy efficiency [3]. 3D printing offers the prospect of rapid prototyping, and new materials can have structural properties that cannot be achieved by other manufacturing methods. Because of the variety of 3D printing techniques that allow new shapes to be created, such as melting material, curing resin, or laser sintering powders, it is possible to design and print virtually any model. The use of 3D printing techniques in modern industry is associated with the achievement of greater dimensional accuracy and shorter prototype construction times [4]. AM can be applied to a wide range of materials including polymers [5], ceramics [6], metals [7], concrete [4], soil [8], and tissues [9]. 3D printing is an umbrella term covering a number of different technologies. According to ISO/ASTM 52900:2021 [10], there are seven different categories of AM processes (ISO/ASTM International, 2021). The most commonly used in modern industry are stereolithography (SLA—1986, 3D System), fused deposition modelling (FDM—1988, Stratasys), laminated object manufacturing (LOM—1991, Helisys), selective laser sintering (SLS—1992, DTM Corporation), and direct metal laser sintering (DMLS—1995, EOS GmbH), Figure 1.

An analysis of the market for thermal insulation solutions points to the rapid growth of 3D printing. By precisely controlling the production process, it is possible to create materials with a complex porous structure that have great potential for use in construction and other industries. However, creating a structural composite with good insulating properties is not easy. Porous structures are characterized by low relative density, large specific surface area, and good mechanical properties.

It is important to note that 3D printing is a relatively new technology that requires further research and testing to optimize and adapt the material to specific needs, such as construction [11,12,13]. Research has focused primarily on investigating the influence of structural parameters on the mechanical properties of materials, such as Young’s modulus and compressive strength [14,15,16]. However, research into the thermal conductivity of porous structures is not comprehensive. A complete understanding of the thermal conductivity of the described structures and the exploitation of their potential in various applications, including thermal insulation, energy storage, or the production of advanced structural materials, is a challenge for engineers, architects, and thermal insulation manufacturers. This process requires an interdisciplinary approach involving physics, chemistry, or materials engineering. In addition, most porous materials tested are homogeneous. However, in engineering applications the loading of porous materials is often very complex and homogeneous porous materials do not meet the application requirements. In building applications, porous materials should not only be lightweight, but also have high strength, fire resistance, and low thermal conductivity [17,18,19]. In porous structures, the relationship between pore geometry and thermal properties can be complicated. Many factors such as shape, size, degree of porosity, and material type can affect thermal conductivity, making the analysis complex.

In the scientific literature, there is work on the study of the thermal insulation properties of prototype bionic multilayer porous materials based on the Kelvin foam model and manufactured using additive SLS technology [11]. The study investigated the effect of layer number, pore diameter, and porosity on the thermal insulation properties of 3D-printed nylon composites. The composites were characterized by an open cell core and sandwich structure for two variations of heat flow (top-to-bottom and bottom-to-top) [11].

Other research has used TPMS structures to create materials with specific properties. Research on porous TPMS structures focuses on finding a configuration that provides the most favorable combination of material properties, i.e., mechanical, thermal, and optical [20,21,22,23,24]. Much analysis has been devoted to the design of a spatial TPMS structure with a gyroid or diamond insert, the shape of which mirrors the bionic structure of the bone interior [20,21,25]. The test results showed that the thermal insulation of the prototype insulating partitions with a gyroidal structure was characterized by good insulation parameters. The lowest value of thermal conductivity and the highest value of thermal resistance of the insulating material were 0.034 W/m·K and 0.586 m^2^·K/W, respectively [21]. In addition to gyroidal and diamond textures, many other textures are available, such as cubic, lattice, triangular, star, linear, concentric, 3D honeycomb, Hilbert curve, Archimedean spiral, octagonal spiral, and others [25,26].

The purpose of subsequent research was to evaluate the thermal and mechanical properties of thermoplastic polyester PET-G (polyethylene terephthalate-glycol) with different microstructure patterns [27]. Thermal tests were carried out in a hot-box test chamber and mechanical properties were assessed using a three-point flexure test. The test results show differences in thermal efficiency of up to 70% and mechanical efficiency of up to 300%. For each geometry, the mechanical and thermal properties were highly correlated with the infill pattern, with improvements in thermal and mechanical properties observed as the infill density increased.

In recent years, the use of natural waste materials has played an important role in the development of thermal insulation technology [12,28]. In [12], cubic samples of biodegradable polylactic acid (PLA) filament [16] were designed with square and hexagonal hole types. The analysis showed that insulation with hexagonal holes had better thermal properties than samples with square holes. Printed composites can be successfully used as thermal insulation materials, for which the lowest values of the thermal conductivity coefficient were 0.023 W/(m·K). The researchers of [28] aimed to compare the thermal performance of a 3D-printed PLA block with a honeycomb block. The analysis assumes that the cavities are filled with natural and recyclable insulation waste, such as (i) wood sawdust, (ii) sheep wool, and (iii) hemp. The research results show that the use of waste materials significantly improves the thermal parameters of 3D prints, reducing the heat transfer coefficient by 57% [28].

The study of porous gradient structures is an area of research that focuses on the analysis, design, and use of materials with variable porosity or structure. A Voronoi diagram can be used to analyze the spatial distribution of pores in a porous material. An important aspect of the research is the study of the thermal properties of structures designed using Voronoi spatial tessellation [29]. The authors of this study analyzed regular and spatial Voronoi gradient structures for thermal protection and the influence of porosity, gradient direction, and heat flow density on the thermal properties of the structure [29]. The results show that the effective thermal conductivity of the spatial Voronoi gradient structure is lower than that of the regular structure. Effective thermal conductivity decreases significantly with increasing porosity.

The study of porous layered structures can lead to innovative solutions in the design of building materials with different properties. Laminated structures have rigid outer layers and a low-density core in the form of closed seals made of a material with air properties, such as foam or periodic structures [30]. The use of honeycomb core panels has received the most research attention [31,32,33]. Honeycomb structures are closed cell structures. They consist of plates or sheets forming the edges of the closure, which can be arranged in triangles, squares, hexagons, or other related shapes, and their unit closures can be repeated in two dimensions to form a solid [34]. Such cores are stiff, lightweight, and absorb high energy under the influence of impacts and shock waves, which is important for many industrial applications [30,35,36,37].

The use of metamaterials also opens up many opportunities for innovative material solutions, particularly in building technology. The structure of metamaterials allows heat flow to be controlled, improving the thermal insulation of buildings and increasing energy efficiency [38].

Another important research issue arising from the use of additive technology is the fire resistance of products. The use of bionics makes it possible to create structures that are optimized not only for thermal efficiency but also for fire resistance. Modern 3D printing techniques make it possible to print integrated structures with more complex patterns, which can improve fire resistance through better distribution and dissipation of heat during a fire. Much research has focused on finite element modelling to predict the fire resistance of 3D-printed bionic-inspired concrete wall panels [39].

**Figure 2:**
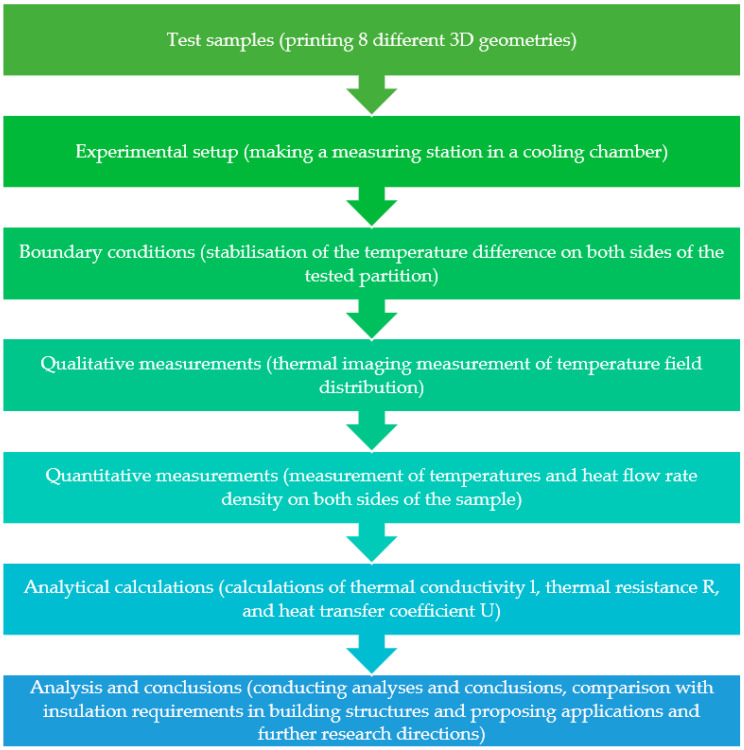
Methodological process used in this study (elaborated by authors).

In conclusion, despite the wide range of research presented in the scientific literature, there is a noticeable lack of more comprehensive analyses of the structural geometry and thermal properties of insulation materials produced using additive technology. The authors of this paper analyzed the influence of the geometry of the inner core of cellular composites produced by stereolithography (SLA) and selective laser sintering (SLS). The main objective of the experimental study was to analyze 3D-printed samples of prototype composites in terms of their thermal insulation and applicability in construction. The flowchart of the research procedure used is shown in Figure 2.

## 2. Materials and Methods

### 2.1. Design and 3D Printing of Multilayer Insulation

Based on the literature review and previous research carried out by the co-author of this article [11,12,20,21,26,40], three-layer composites with different geometries of the inner core structure produced using SLS 3D printing technology were selected for analysis. In addition, to demonstrate the practical application of the composite characterized by the lowest thermal conductivity coefficient, sandwich panels with dimensions larger than those of the SLS samples studied were produced. These panels were produced on a 3D printer DAZZ 3D SLA S130 (Shenzhen, China) using SLA technology.

SLS 3D printing using polymer powder (PA12) sintered by a high energy laser is an innovative technology. It offers considerable dimensional accuracy by sintering fine plastic particles and assembling them in layers. The entire process is based on previously generated cross-sectional data using specialized computer programs, i.e., Solid Edge, Inventor, and other 3D printer software (Rhino 7). One of its advantages is the lack of need for structural supports, which greatly simplifies the manufacturing process [27,41].

SLA is a pioneering technology that uses low-power laser light to cure liquid resins layer by layer. The key feature of this process is its high level of accuracy, which allows even the finest details to be produced with precision. This printing is characterized by precision and considerable speed, due to the use of a powerful laser light source to cure the resin layers [26].

Both 3D printing technologies were chosen for the test samples because of their accuracy, which was a necessary parametric feature of the 3D printing used, given the complex shapes of the composites.

The designed thermal insulation material is a composite consisting of air voids of a given geometry and a skeleton of polymer material and resin.

### 2.2. Geometry of Test Samples

**Figure 3:**
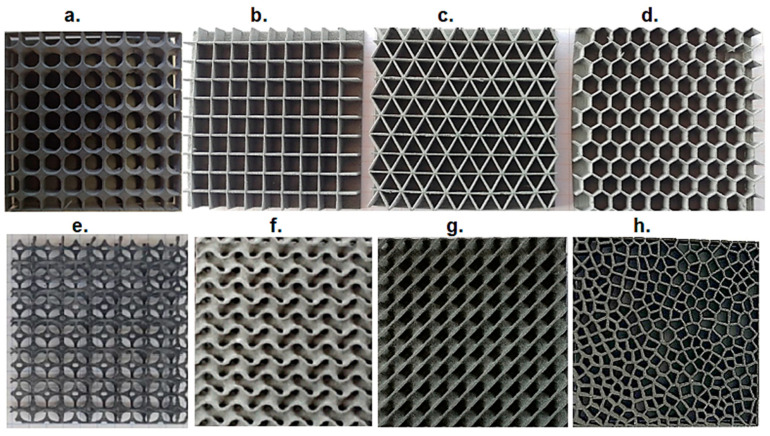
Geometry of structures, 3D SLS-printed test samples: (**a**) circular, (**b**) square, (**c**) triangular, (**d**) hexagonal, (**e**) Kelvin tetrahedron, (**f**) gyroid, (**g**) diamond, (**h**) 2D Voronoi (elaborated by authors).

**Figure 4:**
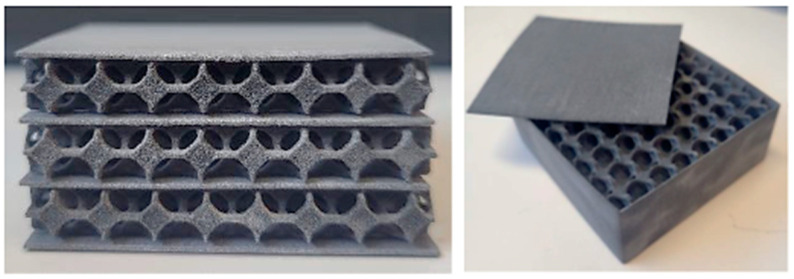
Example of a three-layer cellular composite sample with an inner core structure based on a Kelvin tetrahedral model produced by 3D SLS printing. Based on [12].

The subjects of this study were sandwich composites, three-layer closed-cell (each layer approximately 6.6 mm thick), closed-cell, and open-cell. They had a core structure based on the geometry of a circle, triangle, square, hexagon [12,26,40], Voronoi diagram and the structure of a gyroid, diamond [20,21], and Kelvin tetrahedron [16] (Figure 3). Each sample was characterized by plan dimensions of 60 × 60 mm and a thickness of 20 mm (Figure 4). 

The dimensions of the repeatable module for each structure were as follows: (a) circular, diameter of circle 6 mm, wall thickness t = 0.2 mm; (b) square, side length 6 mm, wall thickness t = 0.2 mm; (c), triangular, height length of the triangle 6 mm, wall thickness t = 0.2 mm; (d) hexagonal, S = 6 mm, t = 0.2 mm; (e) Kelvin tetrahedron, d = 6 mm and *p* = 0.95; (f) gyroidal, t = 0.2 mm; ab = 3π, c = 2π; (g) diamond, t = 0.2 mm; ab = 3π, c = 2π; and (h) 2D Voronoi, number of air cells equal to 500, t = 0.2 mm.

**Figure 5:**
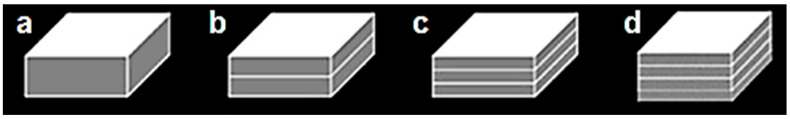
Sketch of 3D-printed cellular composites with different layering: (**a**) 1-layer; (**b**) 2-layer; (**c**) 3-layer; (**d**) 4-layer (elaborated by authors).

**Figure 6:**
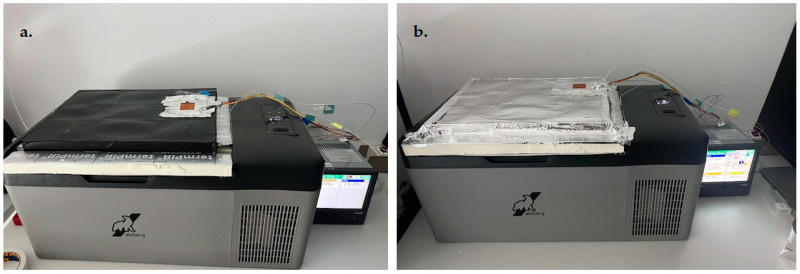
Test stand for measuring thermal properties of structured panels with the geometry of the inner core of the gyroid made using 3D SLA technology; (**a**) four-layer panel with outer black polyethylene film, (**b**) four-layer panel with outer metallized polyethylene film (elaborated by authors).

The SLA process was used to print prototypes of thermal insulation panels for cellular compounds with the lowest thermal conductivity. These were gyroid-shaped core structures for single, double, triple, and quadruple layers (Figure 5), which were additionally coated with an outer layer of black polyethylene film (Figure 6).

### 2.3. Experiments

**Figure 7:**
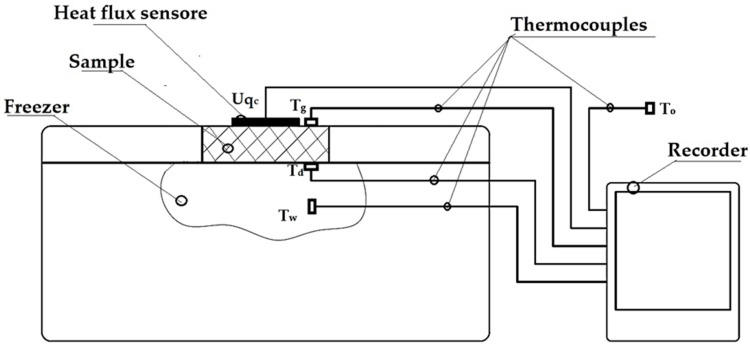
Schematic of the test stand for thermal insulation testing (elaborated by authors).

**Figure 8:**
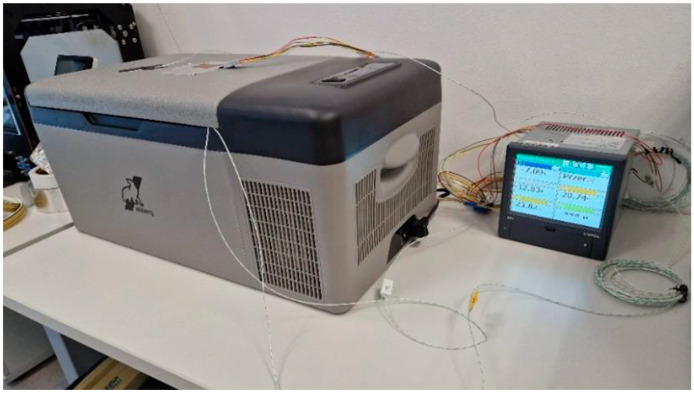
Photograph of the test stand (elaborated by authors).

For each of the described and printed prototype composite samples, the values of the thermal conductivity coefficient λ and the thermal resistance R (for a sample thickness of 20 mm) were experimentally determined. Measurements were carried out according to ISO 9869-1:2014 [42] on an existing test rig at the Department of Energy Conversion Engineering, Faculty of Mechanical and Energy Engineering, Wroclaw University of Science and Technology. A schematic diagram of the test rig is shown in Figure 7 and a photograph of the test rig is shown in Figure 8.

During the measurements, the samples were placed in a hole in the lid of an Aisberg LP15 C15 freezer (MELIS, Poznań, Poland)so that the bottom of the samples was in direct contact with the inside of the freezer and the top with the outside. A frame measuring 340 × 265 × 20 mm was constructed in place of the lid to accommodate samples measuring 60 × 60 × 20 mm. During a test, four samples of different types were placed simultaneously in the area of the freezer lid.

The mechanism of heat flow through the specimen was based on the temperature difference between the environment (outside) and the inside of the freezer. The heat flux density through the insulation under test was measured using an FHF04SC sensor (Hukseflux Thermal Sensors B.V., Delft, The Netherlands) and the data were recorded on a recorder every 0.5 min. During the measurements, temperatures were measured at the following locations: on the outside surface of the sample, on the inside surface of the sample, inside the fridge/freezer, and around the outside of the fridge/freezer (see location of thermocouples in Figure 7). Temperatures outside the sample were assumed to be +20 °C (on the ambient side) and −20 °C (in the refrigerator/freezer compartment) due to the typical operating conditions of thermal insulation of buildings, the food industry, and the transport of frozen foods. The accuracy of the measuring instruments is given in Table 1.

For these boundary conditions, the thermal insulation of the materials was measured at an average sample temperature of 0 °C. The measured values were used to calculate the thermal conductivity coefficients λ and the thermal resistance R. The measured values were recorded after thermal equilibrium had been reached. This state was considered to have been reached when the temperature variation at the surface of the test specimens did not exceed 0.5 °C for successive readings over a period of 1 h. In the experiment carried out, the effect of the geometry of the cellular composite core structure on its thermal conductivity was determined.

In addition to the quantitative measurement of the thermal insulation of the test specimens, the homogeneity of their thermal insulation was qualitatively investigated in the experiment. For this purpose, non-destructive thermal imaging was used to image the uniformity of the temperature field distribution on the warm surface (outer surface) of the sample. A Testo 882 thermal imager with a thermal resolution of 320 × 240 px and a thermal sensitivity of less than 50 m·K with a 32° lens and an IFOV parameter of 1.7 mrad was used for the thermal measurement. The infrared measurement range was between 8 and 14 µm. An emissivity parameter of 0.94 was set for the whole thermogram, as was the case for plastic.

### 2.4. Quantitative Method for Calculating Thermal Parameters

The methodology for quantifying the thermal parameters was based on measuring the electrical voltage and converting it into heat density flux according to Equation (1) specified by the device manufacturer.

(1) $$q=\frac{Uqc}{0.0103},$$

where:

*q*—heat flux density, [W/m^2^];
*Uqc*—voltage of the flowing current, [mV].

At the same time, the temperatures on the top (hot) and bottom (cold) surfaces of the test samples, as well as the air temperature inside and outside the cold chamber, were measured on the test bench. These temperatures were measured using K-type thermocouples. Based on the measured temperatures and the heat flux density during the steady-state phase of heat flow through the sample, the heat transfer coefficient was calculated using Equation (2).

(2) $$\lambda =\frac{d\cdot q}{T_{g}-T_{d}},$$

where:

*λ* is the design thermal conductivity of the material, [W/m·K];
*d* is the thickness of the test sample, [m];
*q* is the heat flux density, [W/m^2^];
*T_g_* is the temperature of the upper surface of the sample, [°C];
*T_d_* is the temperature of the lower surface of the sample, [°C].

Subsequently, the heat transfer coefficients U were estimated for the material thicknesses determined according to the methodology specified in ISO 6946 [43] as well as for the homogeneous material partitions. The calculations assumed a horizontal direction of heat transfer, as for vertical external partitions (walls). This assumption allowed for the selection of appropriate thermal resistance coefficients for the internal air layers *R_si_* = 0.13 and the external *R_se_* = 0.04. The U-value was determined according to Equation (3).

(3) $$U=\frac{1}{R_{si}+\sum_{i}\frac{d_{i}}{\lambda_{i}}+R_{se}}$$

where:

*U* is the thermal transmittance, [W/m^2^·K];
*R_si_* is the internal surface resistance, [m^2^·K/W];
*R_se_* is the external surface resistance, [m^2^·K/W];
*d_i_* is the thickness of the material layer (i) in the component, [m];
*λ_i_* is the design thermal conductivity of the material layer (i), [W/m·K].

## 3. Results

### 3.1. Qualitative Evaluation of Thermal Insulation Using Thermal Imaging

**Figure 9:**
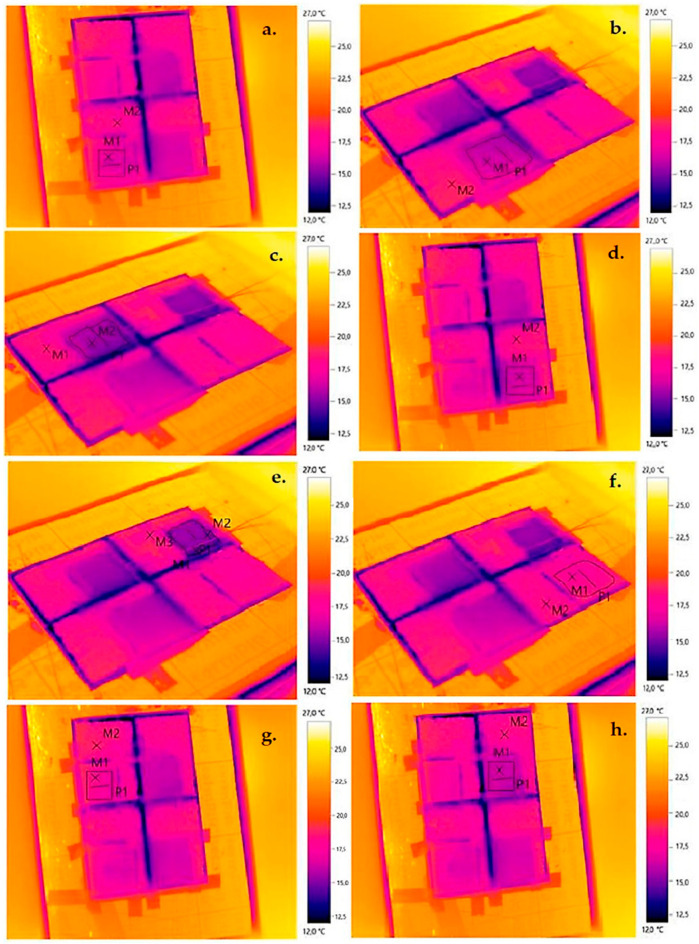
Thermograms for individual structures in SLS printing technology: (**a**) circular, (**b**) square, (**c**) triangular, (**d**) hexagonal, (**e**) Kelvin tetrahedral, (**f**) gyroidal, (**g**) diamond, (**h**) 2D Voronoi (elaborated by authors).

**Figure 10:**
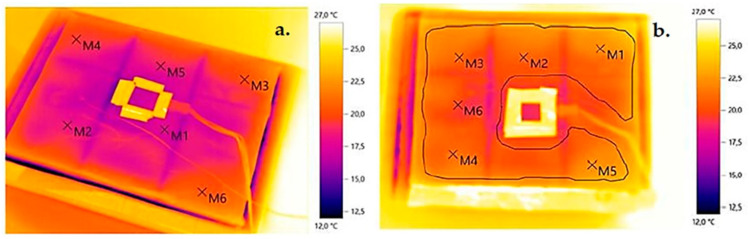
Thermograms for structures printed using SLA technology: (**a**) single-layer gyroid, (**b**) double-layer gyroid (elaborated by authors).

The qualitative results of the uniformity of the temperature field distribution on the external (warm) surface for the thermal imaging tests performed are shown below (Figure 9—for the SLS print and Figure 10—the SLA print for the gyroid). 

### 3.2. Quantitative Evaluation of Thermal Insulation by Means of Heat Density Measurement

The quantitative evaluation of the thermal insulation properties of the tested composites, i.e., the determination of the thermal conductivity coefficient, was carried out according to the calculation method described in Section 2.4. The measured and calculated values are presented in Table 2 and Table 3 as results averaged over 3 replicates for each type of specimen analyzed. The measured values were taken at the steady state of heat conduction, i.e., approximately 48 h after the start of the sample test. Table 4 and Table 5 show the measurement errors of the quantitative calculations.

Statistical evaluations were conducted utilizing functionalities provided within the STATISTICA 13 software (TIBCO Statistica, Palo Alto, CA, USA). A significance threshold of *p* ≤ 0.05 was used (a widely accepted criterion in thermal insulation). For the values obtained from the experimental data of the composites printed with 3D SLS technology, the position and dispersion measures were first determined, and their aggregated results are presented in Table 6.

The λ thermal conductivity values ranged between 0.0349 and 0.0781 W/(m·K), with an average of 0.0565 W/(m·K) and a standard deviation of 0.0116 W/(m·K). Approximately half of the tested samples exhibited values of 0.0540 W/(m·K) or lower. On the thermal resistance scale R, the findings ranged from 0.2690 to 0.5100 (m^2^·K)/W, with an average of 0.3625 (m^2^·K)/W and a deviation of 0.0671 (m^2^·K)/W. About half of the samples showed results of 0.3650 (m^2^·K)/W or lower. U-values varied from 0.9890 to 1.5810 W/(m·K), with a mean of 1.2713 W/(m·K) and a standard deviation of 0.1612 W/(m·K). Half of the tested samples exhibited values of 1.29 W/(m·K) or lower. The observed skewness and kurtosis values suggest that most of the sample results clustered around the mean. Subsequently, an assessment was made to determine the impact of the input quantities on the output quantities in the experiment. A one-way ANOVA analysis was employed for this purpose. The results are detailed in Table 7, Table 8 and Table 9. 

**Figure 11:**
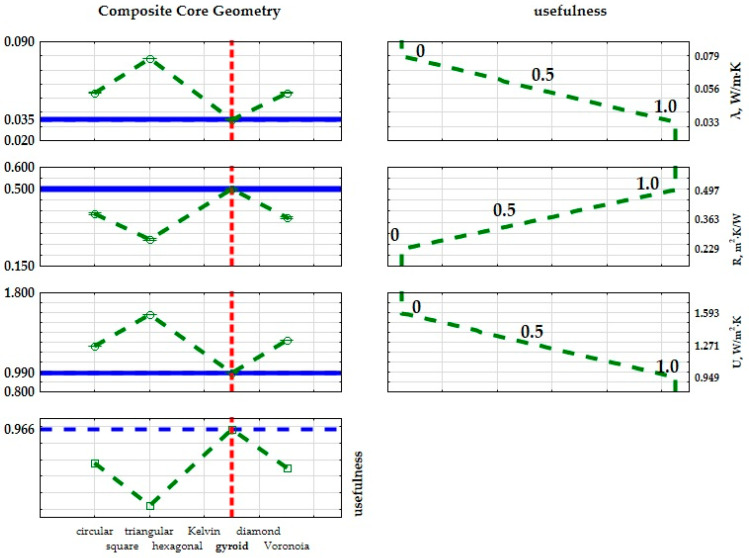
Graphical interpretation of the optimization of composite structures based on the thermal conductivity coefficient (λ), thermal resistance coefficient (R), and thermal transmittance coefficient (U) determined from the SLS 3D-printed samples.

**Figure 12:**
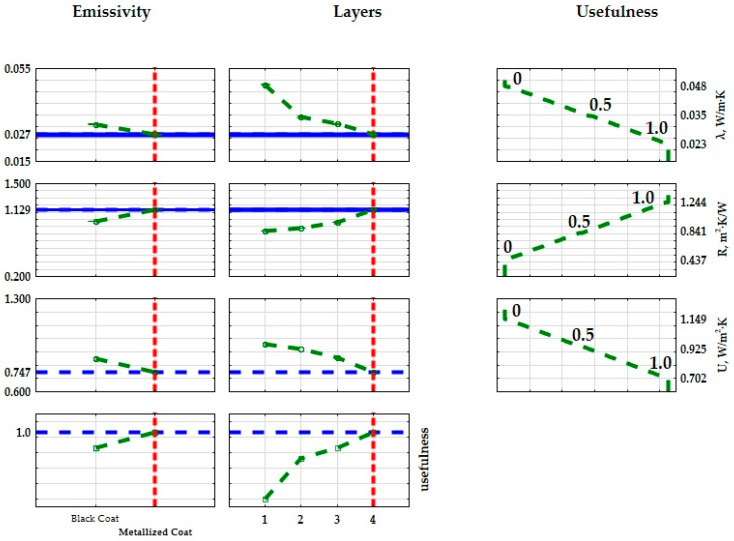
Graphical interpretation of the optimization of composite structures based on the thermal conductivity coefficient (λ), thermal resistance coefficient (R), and thermal transmittance coefficient (U) determined from the SLA 3D-printed samples.

The provided *p*-values, below 0.05 (as shown in the final column of the table), signify a notable impact of the inner core type on the thermal conductivity coefficient, thermal resistance, and transmission coefficient of the examined composites. The results from the analysis of variance (Table 7, Table 8 and Table 9) reveal the impact of the inner core geometry on the resultant values of the thermal conductivity coefficient (λ), thermal resistance coefficient (R), and heat transfer coefficients (U). In essence, the optimal insulating characteristics among the fabricated composites are attained by utilizing a three-layer cellular structure composite featuring a gyroid inner core geometry. Each input factor is individually fine-tuned to improve performance. To refine the structure of the produced composites, practical lower, middle, and upper bounds for the thermal conductivity coefficients were computed, aiming for the most favorable thermal properties suitable for construction and industrial applications. Practical values were assigned to these bounds: for the ‘max criterion’, the upper limit was set at 1.0 and the lower limit at 0, with a linear variation between them resulting in an intermediate value of 0.5. The extreme mean values of the thermal conductivity coefficient are shown in Figure 11 and Figure 12. Given the relatively uniform distribution of the thermal conductivity coefficients, the criterion to identify the best insulating properties of the fabricated composite was based on the lowest value of the thermal conductivity coefficient. Therefore, the most effective material exhibited a structure with superior thermal insulation properties.

In the next step of values from the experimental data obtained for composites printed with 3D SLA technology, the positions and dispersion measures were first determined, and their aggregated results are shown in Table 10.

The λ values ranged from 0.0266 to 0.0481 W/(m·K), with an average of 0.0352 W/(m·K) and a standard deviation of 0.0063 W/(m·K). Approximately half of the tested samples exhibited values of 0.0341 W/(m·K) or lower. On the R thermal resistance scale, the results varied between 0.53 and 1.193 (m^2^·K)/W, with an average of 0.8405 (m^2^·K)/W and a deviation of 0.2017 (m^2^·K)/W. About half of the samples displayed results of 0.8854 (m^2^·K)/W or lower. U-values ranged from 0.7466 to 1.09 W/(m·K), with a mean of 0.9254 W/(m·K) and a standard deviation of 0.1117 W/(m·K). Half of the tested samples exhibited values of 0.9129 W/(m·K) or lower. The skewness of the results suggests that the majority of samples were clustered around the mean. The subsequent step involved assessing whether the input quantities in the experiment significantly influenced the output quantities. A two-factor ANOVA analysis was conducted for this purpose. The results are summarized in Table 11, Table 12 and Table 13.

The extreme average of the thermal conductivity values is shown in Figure 12. Since the thermal conductivity values are relatively homogeneous, the lowest thermal conductivity value was determined as a criterion to determine the best insulating properties of the plastered composite. The most useful material had a structure with the best thermal insulation properties.

## 4. Discussion

The preliminary results indicate a significant correlation between geometric differences in air voids in the 3D-printed composite parts and their thermal resistance. The results suggest that 3D printing geometry tuning may be a key factor in optimizing 3D-printed composites for thermal insulation purposes. Qualitative thermal imaging studies indicate the absence of thermal bridging in repetitive module bonding of 3D-printed structures. The temperature field distribution recorded by the thermal imaging camera can be considered uniform (differences of less than 2 °C).

**Figure 13:**
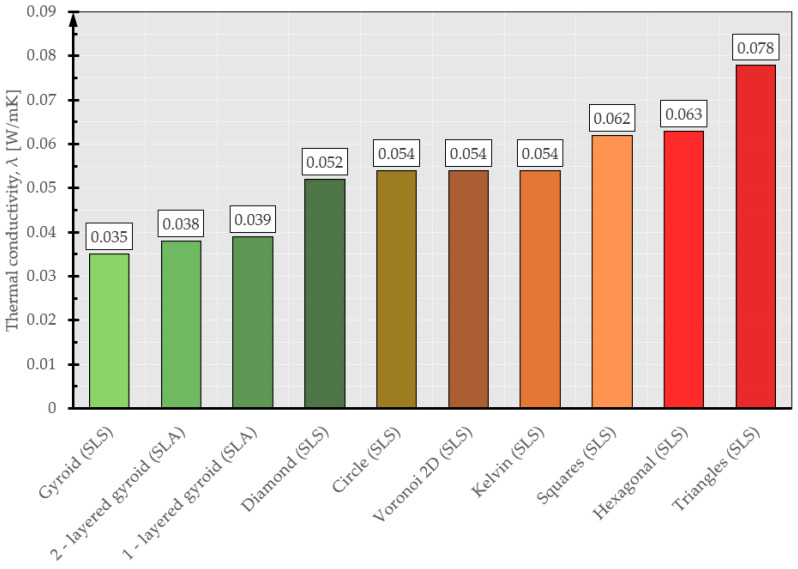
The coefficients of thermal conductivity for 3D-printed geometries.

Comparing the thermal parameters obtained (thermal conductivity coefficient) with typical building materials [44,45], it can be concluded that the proposed 3D-printed geometries have similar or even better (lower) thermal conductivity coefficients than the materials used as typical thermal insulation in buildings (polystyrene, mineral wool). Figure 13 shows the thermal conductivity coefficients for the 3D-printed geometries. Geometries with λ values less than 0.05 can be considered good thermal insulation materials. 

**Figure 14:**
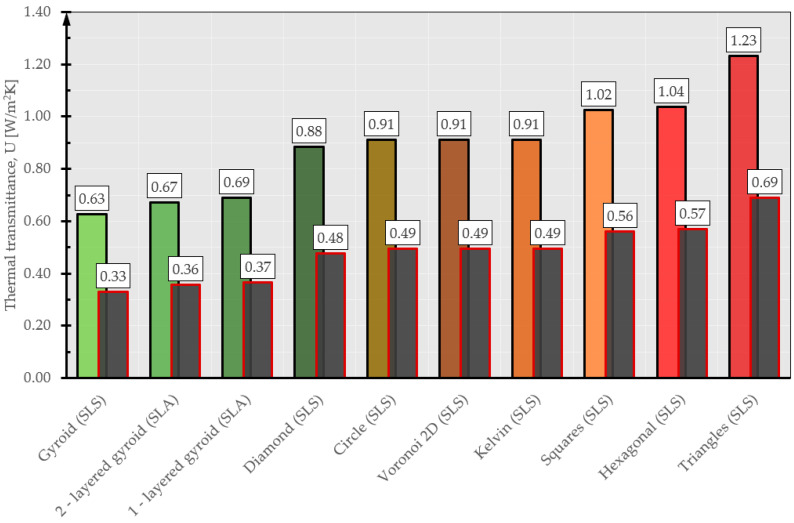
Estimated heat transfer coefficients for various 3D geometries. Black border: thickness 50 mm, red border: thickness 100 mm.

An interesting proposal for the use of 3D-printed materials with optimized geometry in terms of thermal insulation is the production of window frames. In the case of windows, their thermal insulation depends on both the glazing set and the window frame. Typical window frames are a few tens of millimeters thick and range in thickness from 50 to about 100 mm [46]. For such geometries, the heat transfer coefficient (U_f_) of typical PVC window frames ranges from 0.8 to 1.4 W/m^2^·K [46]. In the case of using 3D-printed geometries to fill window frames or producing entire window frames using 3D printing technology, the estimated U_f_ cofactor would be approximately 0.33 W/m^2^·K (for a 100 mm thick frame) to 0.63 W/m^2^·K (for a 50 mm thick frame) for gyroid geometries (Figure 14). These values are estimates based on the thermal conductivity and thickness of the material and do not take into account the variation of thermal conductivity with sample thickness and number of layers.

**Figure 15:**
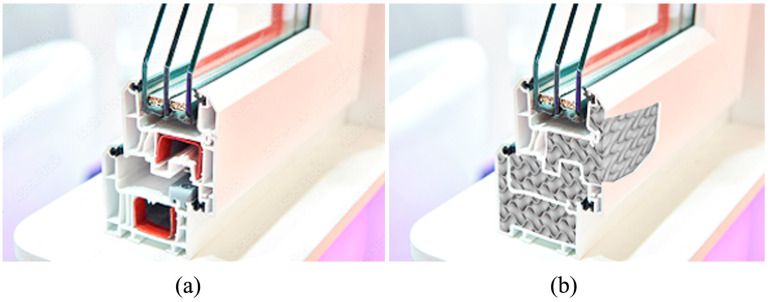
Cross section of a window frame with an example of using 3D-printed geometries to make window frames: (**a**) a typical window frame, (**b**) a 3D-printed frame.

Figure 15 shows a possible example of filling a window frame with 3D-printed geometry.

With the appropriate thickness of the proposed 3D-printed materials (i.e., about 15–20 cm), these materials can also be used as thermal insulation for opaque building partitions such as exterior walls, ceilings, and roofs. Such partitions will meet today’s stringent requirements for thermal insulation and energy efficiency.

## 5. Conclusions

On the basis of the research and analyses carried out, the following conclusions were drawn:

1. Thermograms confirmed the uniform distribution of the temperature field over the entire surface of the printed test samples.
2. The best (lowest) thermal conductivity is demonstrated by a 3D-printed structure in the form of a gyroid with a thermal conductivity coefficient of 0.035 W/m^2^·K.
3. The 3D-printed gyroidal structure has thermal insulation properties similar to those of typical thermal insulation materials used in construction, such as mineral wool and polystyrene.
4. Increasing the number of layers (in the same material thickness) significantly reduces the thermal conductivity of the printed structure. This is due to the smaller airspaces, which reduce the amount of heat transferred by convection and radiation.
5. The proposed gyroid geometry is expected to be used to print the filling in the window frames, which will increase the stiffness and strength of the frames while reducing thermal conductivity (greater energy efficiency).

The integration of 3D printing into thermal insulation composites can have far-reaching implications for the construction industry. Customized solutions of 3D-printed composite geometries to specific thermal insulation requirements can increase and optimize energy efficiency and reduce thermal losses in various applications.

Despite the promising potential of 3D printing in thermal insulation, challenges such as material selection, scalability, and cost effectiveness need to be addressed. Future research should focus on improving the printing parameters, exploring new composite materials, and developing standardized testing methods. In the case of the proposed application of 3D-printed window frames, a key issue that requires further research is the verification of the mechanical strength of such a product.

The article demonstrates the potential of 3D printing technology in the field of thermal insulation composites. Experimental studies of the effect of geometry on thermal resistance show that adjusting the appropriate geometry of the air voids (spaces between the printed frame) using 3D printing is key to optimizing the insulating properties of these materials. As research in this area continues, the integration of 3D printing is poised to revolutionize the approach to thermal insulation, offering sustainable and efficient solutions for a variety of applications, including printed aerospace structures.

## Figures and Tables

**Table 1:** The accuracy of the measuring instruments.

Measuring Device	Accuracy
K-type thermocouple	0.1 K
FHF04SC heat flux sensor	11 μV/(W/m^2^)
Vernier caliper	0.05 mm

**Table 2:** Measured temperatures and heat flux densities and calculated values for thermal conductivity, thermal resistance, and heat transfer coefficient of SLS 3D-printed samples.

Type Geometry	*d* [mm]	*V_qc_* [mV]	*q* [W/m^2^]	*T_g_* [°C]	*T_d_* [°C]	*λ* [W/m·K]	*R* [m^2^·K/W]	*U* [W/m^2^·K]
Gyroid	20	0.38	37.50	15.1	−3.80	0.035	0.50	0.99
Diamond	20	0.46	44.70	13.7	−3.40	0.052	0.38	1.19
Circle	20	0.40	46.60	15.1	−3.21	0.054	0.39	1.13
2D Voronoi	20	0.53	51.70	15.7	−3.40	0.054	0.37	1.26
Kelvin	20	0.49	48.10	13.0	−4.50	0.054	0.36	1.32
Aquares	20	0.51	49.60	12.1	−2.60	0.062	0.29	1.29
Hexagonal	20	0.48	46.80	13.0	−3.10	0.063	0.34	1.31
Triangles	20	0.60	59.40	12.5	−3.50	0.078	0.27	1.29

**Table 3:** Measured temperatures and heat flux densities and calculated values for thermal conductivity, thermal resistance, and heat transfer coefficient of SLA 3D-printed samples.

Type Panel	*d* [mm]	*V_qc_* [mV]	*q* [W/m^2^]	*T_g_* [°C]	*T_d_* [°C]	*λ* [W/m·K]	*R* [m^2^·K/W]	*U* [W/m^2^·K]
four-layer panel with outer metallized	40	0.23	21.85	19.32	−5.41	0.026	1.129	0.75
three-layer panel with outer metallized	40	0.26	25.15	19.33	−4.9	0.031	0.964	0.85
two-layer panel with outer metallized	40	0.28	27.57	19.27	−4.81	0.034	0.876	0.92
one-layer panel with outer metallized	40	0.29	28.25	17.79	−5.76	0.048	0.832	0.96
four-layer panel with outer black	40	0.23	22.14	19.7	−1.79	0.031	0.968	0.85
three-layer panel with outer black	40	0.27	26.21	19.6	−3.87	0.034	0.894	0.91
two-layer panel with outer black	40	0.42	40.97	20.3	−1.60	0.038	0.53	1.08
one-layer panel with outer black	40	0.41	39.40	18.5	−2.60	0.039	0.53	1.09

**Table 4:** Measurement errors for quantitative calculations of SLS 3D-printed samples.

Type Geometry	Δ*d* [mm]	Δ*T* [°C]	Δ*V_qc_* [mv]	Δ*q* [W/m^2^]	Δ*λ* [W/m·K]	Δ*R* [m^2^·K/W]
Gyroid	0.1	0.1	0.023	2.21	0.00492	0.02365
Diamond	0.1	0.1	0.026	2.55	0.00677	0.01766
Circle	0.1	0.1	0.027	2.65	0.00573	0.02023
2D Voronoi	0.1	0.1	0.032	3.10	0.00728	0.01611
Kelvin	0.1	0.1	0.026	2.53	0.00623	0.01896
Aquares	0.1	0.1	0.028	2.72	0.00776	0.01559
Hexagonal	0.1	0.1	0.029	2.79	0.00700	0.01693
Triangles	0.1	0.1	0.033	3.17	0.00822	0.01448

**Table 5:** Measurement errors for quantitative calculations of SLA 3D-printed samples.

Type Geometry	Δ*d* [mm]	Δ*T* [°C]	Δ*V_qc_* [mv]	Δ*q* [W/m^2^]	Δ*λ* [W/m·K]	Δ*R* [m^2^·K/W]
four-layer panel with outer metallized	0.1	0.1	0.026	2.56	0.00691	0.01745
three-layer panel with outer metallized	0.1	0.1	0.032	3.14	0.00748	0.01650
two-layer panel with outer metallized	0.1	0.1	0.026	2.57	0.00642	0.01886
one-layer panel with outer metallized	0.1	0.1	0.028	2.75	0.00776	0.01560
four-layer panel with outer black	0.1	0.1	0.033	3.10	0.00815	0.01458
three-layer panel with outer black	0.1	0.1	0.029	2.72	0.00710	0.02019
two-layer panel with outer black	0.1	0.1	0.023	2.25	0.00448	0.02560
one-layer panel with outer black	0.1	0.1	0.027	2.60	0.00587	0.01987

**Table 6:** Descriptive statistics of thermal conductivity coefficient (λ), thermal resistance (R), and heat transmission coefficient (U) (*Min*—minimum; *Max*—maximum; *M*—mean; *SD*—standard deviation; *Me*—median; *Sk*—skewness; *K*—kurtosis) of the SLS 3D-printed samples.

	*M*	*Me*	*Min*	*Max*	*SD*	*Sk*	*K*
λ, W/(m·K)	0.0565	0.0540	0.0349	0.0781	0.0116	0.0274	0.5593
R, (m^2^·K)/W	0.3625	0.3650	0.2690	0.5100	0.0671	0.6951	0.4881
U, (W/m^2^·K)	1.2713	1.2900	0.9890	1.5810	0.1612	0.1556	0.5222

**Table 7:** Quantitative evaluation of the main effects—identifying the effect of a statistically significant input factor on the dependent variable λ of SLS 3D-printed samples.

Symbol That Identifies the Input Factors	*SS*	*df*	*MS*	*F*	*p*
absolute term	0.076614	1	0.07661	7,661,400	0.00
composite core geometry	0.003108	7	0.00044	44,400	0.00
error	0.0000002	16	0.00000001		

**Table 8:** Quantitative evaluation of the main effects—identifying the effect of a statistically significant input factor on the dependent variable R of SLS 3D-printed samples.

Symbol That Identifies the Input Factors	*SS*	*df*	*MS*	*F*	*p*
absolute term	3.15375	1	3.15375	235,794.4	0.00
composite core geometry	0.10305	7	0.01472	1100.7	0.00
error	0.00021	16	0.000014		

**Table 9:** Quantitative evaluation of the main effects—identifying the effect of a statistically significant input factor on the dependent variable U of SLS 3D-printed samples.

Symbol That Identifies the Input Factors	*SS*	*df*	*MS*	*F*	*p*
absolute term	38.78330	1	38.78329	33,242,824.3	0.00
composite core geometry	0.59723	7	0.085317	73,129.6	0.00
error	0.000019	16	0.0000012		

**Table 10:** Descriptive statistics of thermal conductivity coefficient (λ), thermal resistance (R), and heat transmission coefficient (U) (*Min*—minimum; *Max*—maximum; *M*—mean; *SD*—standard deviation; *Me*—median; *Sk*—skewness) of the SLA 3D-printed samples.

	*M*	*Me*	*Min*	*Max*	*SD*	*Sk*
λ, W/(m·K)	0.0352	0.0341	0.0266	0.0481	0.0063	0.8214
R, (m^2^·K)/W	0.8405	0.8854	0.5300	1.1293	0.2017	0.5485
U, (W/m^2^·K)	0.9254	0.9129	0.7466	1.0900	0.1117	0.1671

**Table 11:** Quantitative evaluation of both main effects and interaction effects involved in identifying the influence of prominent and statistically significant input factors on the dependent variable λ of the SLA 3D-printed samples (n—number of layers, ε—emissivity).

Symbol That Identifies the Input Factors	*SS*	*df*	*MS*	*F*	*p*
absolute term	0.0297	1	0.0297	501,941.2	0.00
ε	0.000001	1	0.000001	17.8	0.00
n	0.000717	3	0.00024	4030.6	0.00
ε × n	0.000183	3	0.000061	1031.8	0.00
error	0.000001	16	0.000000		

**Table 12:** Quantitative evaluation of both main effects and interaction effects involved in identifying the influence of prominent and statistically significant input factors on the dependent variable R of SLA 3D-printed samples (n—number of layers, ε—emissivity).

Symbol That Identifies the Input Factors	*SS*	*df*	*MS*	*F*	*p*
absolute term	16.9559	1	16.9559	11,795,902.3	0.00
ε	0.2897	1	0.28966	202,344.6	0.00
n	0.5729	3	0.19097	133,124.7	0.00
ε × n	0.0734	3	0.02448	17,149.6	0.00
error	0.000	16	0.0000		

**Table 13:** Quantitative evaluation of both main effects and interaction effects involved in identifying the influence of prominent and statistically significant input factors on the dependent variable U of the SLA 3D-printed samples (n—number of layers, ε—emissivity).

Symbol That Identifies the Input Factors	*SS*	*df*	*MS*	*F*	*p*
absolute term	20.5507	1	20.5507	7,051,659.2	0.00
ε	0.07442	1	0.0744	26,038.5	0.00
n	0.20376	3	0.0679	23,153.5	0.00
ε × n	0.00920	3	0.0031	1037.6	0.00
error	0.0000	16	0.0000		

## Data Availability

Data are contained within the article.
